# Rare cases of Guillain-Barré syndrome after COVID-19 vaccination, Germany, December 2020 to August 2021

**DOI:** 10.2807/1560-7917.ES.2023.28.24.2200744

**Published:** 2023-06-15

**Authors:** Helmar C Lehmann, Doris Oberle, Brigitte Keller-Stanislawski, Thorsten Rieck, Renz Streit

**Affiliations:** 1 Department of Neurology, Klinikum Leverkusen, Faculty of Medicine, University of Cologne and University Hospital Cologne, Cologne, Germany; 2 Division of Safety of Biomedicines and Diagnostics, Paul-Ehrlich Institute, Federal Institute for Vaccines and Biomedicines, Langen, Germany; 3 Robert Koch Institute, Department of Infectious Disease Epidemiology, Berlin, Germany

**Keywords:** Guillain-Barré syndrome, neuritis, facial paresis, COVID-19, vaccination, adverse effect

## Abstract

**Background:**

Guillain-Barré syndrome (GBS) has been associated with vaccination against COVID-19.

**Aim:**

We aimed to compare clinical characteristics and analyse excess GBS cases following administration of different COVID-19 and influenza vaccines in Germany versus the expected numbers estimated from pre-pandemic background incidence rates.

**Methods:**

We analysed safety surveillance data reported to the German national competent authority between 27 December 2020 and 31 August 2021. GBS cases were validated according to Brighton Collaboration (BC) criteria. We conducted observed vs expected (OvE) analyses on cases fulfilling BC criteria levels 1 to 4 for all four European Medicines Agency-approved COVID-19 vaccines and for influenza vaccines.

**Results:**

A total of 214 GBS cases after COVID-19 vaccination had been reported, of whom 156 were eligible for further analysis. Standardised morbidity ratio estimates 3–42 days after vaccination were 0.34 (95% confidence interval (CI): 0.25–0.44) for Comirnaty, 0.38 (95% CI: 0.15–0.79) for Spikevax, 3.10 (95% CI: 2.44–3.88) for Vaxzevria, 4.16 (95% CI: 2.64–6.24) for COVID-19 Vaccine Janssen and 0.60 (95% CI: 0.35–0.94) for influenza vaccines. Bilateral facial paresis was reported in 19.7% and 26.1% of the 156 GBS cases following vaccination with Vaxzevria and COVID-19 Vaccine Janssen, respectively, and only in 6% of cases exposed to Comirnaty.

**Conclusion:**

Three and four times more GBS cases than expected were reported after vaccination with Vaxzevria and COVID-19 Vaccine Janssen, respectively, therefore GBS might be an adverse event of vector-based vaccines. Bifacial paresis was more common in cases with GBS following vaccination with vector-based than mRNA COVID-19 vaccines.

**Key public health message**

**What did you want to address in this study?**

Guillain–Barré-Syndrome (GBS) is an autoimmune condition that can occur after infection or, very rarely, vaccination (incidence: 1.77/100,000 person-years). We wanted to know whether GBS was induced by some COVID-19 vaccines and if yes, whether it was a specific clinical form of GBS.

**What have we learnt from this study?**

Three times and four times more GBS cases than expected were reported after vaccination with the vector-based vaccines (Vaxzevria and COVID-19 Vaccine Janssen) respectively. For COVID-19 mRNA vaccines (Comirnaty and Spikevax) and for influenza vaccines, the number of reported cases did not exceed the number of expected cases.

**What are the implications of your findings for public health?**

Although rare, GBS could be an adverse event after vaccination with vector-based COVID-19 vaccines. Bifacial paresis was more common in cases with GBS following vaccination with vector-based than mRNA-based COVID-19 vaccines.

## Introduction

Guillain–Barré syndrome (GBS) is an autoimmune-mediated polyradiculoneuropathy that is triggered by antecedent infections [1]. Several case series and cohort studies indicate that GBS, including Miller Fisher syndrome (MFS), may also be a rare adverse event of the vector-based COVID-19 vaccines Vaxzevria (ChAdOx1 nCoV-19, AstraZeneca, Cambridge, United Kingdom (UK)) [2-5] and COVID-19 Vaccine Janssen (Ad26.COV2.S, Janssen-Cilag International NV, Beerse, Belgium) [6,7]. Notably, a variant of GBS, clinically characterised by facial diplegia with paraesthesia and absent or only minor motor deficits (FDP) [8-11], has been observed in unusual frequency following administration of vector-based COVID-19 vaccines. Since the FDP variant occurs usually in less than 5% of GBS cohorts [1,12], it raises the question whether bilateral facial paresis with or without motor deficit might be a characteristic clinical phenotype of GBS after COVID-19 vaccination.

We here analysed data on adverse events associated with vaccination with the aim to determine (i) whether there were excess GBS cases following administration of any of the four COVID-19 vaccines in Germany and (ii) whether these cases may have a different clinical presentation, i.e. bifacial paresis or FDP.

## Methods

### Reporting system and data collection

The Paul-Ehrlich-Institute, the German national competent authority for vaccines and biomedicines, collects and evaluates data on all suspected cases of adverse reactions or vaccine complications in Germany provided by (i) healthcare professionals, (ii) consumers or (iii) marketing authorisation holders in the country.

Reports of suspected side effects after vaccination with vaccines (COVID-19, influenza and other) were received via the public health authorities in accordance with the Infection Protection Act [13]. Physicians are legally obliged to report vaccination complications, i.e. health complaints that go beyond the usual extent of a vaccination reaction and are not evidently due to other causes, by name to the competent public health department, which in turn reports them immediately and in pseudonymised form (i.e. without providing the patient's name and address) to the German national competent authority. In addition, the national competent authority receives reports from the drug commissions of pharmacists and physicians, since pharmacists and physicians have a professional obligation to report suspected cases of adverse drug reactions. According to the German Medicines Act, marketing authorisation holders have an obligation to report to the European adverse drug reaction database EudraVigilance. The reports from Germany go from there to the national competent authority.

In addition, healthcare professionals and vaccinated persons or their relatives can report directly to the German national competent authority. Reports can be made by post, e-mail, telephone or electronically via the online reporting portal (www.nebenwirkungen.bund.de). At the German national competent authority, identical reports from different sources are combined into one case. According to the German Medicines Act, the national competent authority is obliged to report reports of suspected adverse drug reactions electronically at certain intervals, pseudonymised and in an internationally standardised format, to the joint EudraVigilance database at the European Medicines Agency, to which every regulatory authority in the European Union has access. 

The spontaneous reporting system for adverse drug reactions after vaccination against COVID-19 and influenza is the same.

Spontaneous reports of suspected side effects are saved in a relational database. Medical Dictionary for Regulatory Activities Terminology (MedDRA) coding is performed by trained personnel (medical documentation assistants) according to the latest coding rules using the latest version of MedDRA. We searched the database from the first licensure of the COVID-19 vaccines on 21 December 2020 until 31 August 2021, using the following MedDRA terms: Standardised MedDRA Queries ‘Guillain–Barré syndrome’ (narrow) and the following preferred terms (PTs): ‘axonal and demyelinating polyneuropathy’, ‘autoimmune demyelinating disease’, ‘acute polyneuropathy’, ‘Bell's phenomenon’, ‘diplegia’, ‘facial paralysis’, ‘facial paresis‘.

### Guillain–Barré syndrome case definition and level of diagnostic certainty

Two physicians (HL and RS) reviewed and validated the reports according to the internationally accepted GBS case definition of the Brighton Collaboration (BC) [14]. Level 1 reflects the highest level of diagnostic certainty, and levels 2 and 3 reflect lower levels of diagnostic certainty. In case of insufficient information, additional information was requested from the reporting physicians using a paper-based questionnaire or as medical reports from reporting consumers if contact details were available. Reports of GBS/MFS that did not correspond to levels 1 to 3 and for which complete information on clinical symptoms was not yet available were assigned level 4 of diagnostic certainty. Level 5 reflected the exclusion of GBS/MFS. At the beginning of the vaccination campaign in Germany, the minimum age for which COVID-19 vaccines were available was 16 years (Comirnaty, BNT162b2, BioNTech-Pfizer, Mainz, Germany/New York, United States (US)). From 31 May 2021 and, respectively, 23 July 2021, mRNA-vaccines Comirnaty and Spikevax (mRNA-1273, Moderna, Cambridge, US) could also be used in individuals 12 years and older. Vaxzevria and COVID-19 Vaccine Janssen were available for persons 18 years and older.

### Negative controls 

We reviewed GBS cases following influenza vaccination for the time period from 1 January 2020 to 31 March 2021 as a negative control. These cases were also sourced from the spontaneous reporting system in Germany with the same reporting requirements for cases following COVID-19 vaccination. During the study period, several tetravalent influenza vaccines of different vaccine types (including one high-dose inactivated influenza vaccine) were available for use in Germany for the influenza season 2020/21. 

### Statistical analysis

The observed vs expected (OvE) analysis compares the frequency of adverse events reported to the national competent authority after vaccination with the statistically random and expected frequencies in a comparable (unvaccinated) population, taking into account different time windows. When the number of reports for an event after vaccination is significantly higher than expected, the national competent authority assumes a safety signal, which should then be further investigated in additional studies. A standardised morbidity ratio (SMR) < 1 with an upper 95% confidence interval (CI) < 1 indicates that significantly fewer reports than expected were recorded, whereas an SMR > 1 with a lower 95% CI > 1 indicates that significantly more reports than expected were recorded. We set the level of significance to α = 0.05.

For the vaccine and age-specific OvE analyses, we included GBS cases who fulfilled the criteria of BC levels of diagnostic certainty 1 to 4 reported to the national competent authority until 31 August 2021 with symptom onset after vaccination and with a known time interval between vaccination and first symptoms (time to onset (TTO)) according to the method published by von Kries et al. [15]. Exposure to the four COVID-19 vaccines was determined based on data from the digital immunisation monitoring system (Digitales Impfquotenmonitoring (DIM)) and data from the practice-based sector, which the national competent authority kindly receives from the Robert-Koch Institute, the national public health institute. In contrast to the vaccination centres, which transmitted individual vaccination records to the DIM, the practices were not connected to the DIM (for technical reasons) but reported vaccination data aggregated by COVID-19 vaccine. For the DIM exposure data, the Robert-Koch Institute provided the national competent authority with a stratification of the doses vaccinated by the cut-off date (31 August 2021) according to vaccine and age group. For exposure data from the practice-based setting, we used data from the Robert-Koch Institute aggregated by vaccine. Because the data from physicians in private practice do not include information on the age of vaccinees, data from a representative group of physicians in private practice were used to determine the vaccine-related age [16]. The resulting vaccine-related age distribution was then projected onto the aggregate data stratified by vaccine that the Robert-Koch Institute receives from physicians of private practices, i.e. we used the vaccine-related age distribution that we found in the representative sample for the aggregated data from the private sector.

The exposure to influenza vaccines (number of doses administered) from January 2020 to March 2021 was calculated according to Siedler et al. [17].

We used age-specific prepandemic background incidence rates for GBS from Denmark published by Levison et al. to calculate expected frequencies as there were no data for Germany of comparable quality and the population structures of the two countries are very similar [18]. Within the scope of the primary analysis, the risk window for symptoms onset was 3–42 days after vaccination.

### Sensitivity analysis

We repeated the OvE analyses including only GBS cases that met BC Level 1 to 3. Two further risk windows for symptoms onset were evaluated: 3–14 days and 3–30 days after vaccination.

All statistical analyses were conducted using SAS, version 9.4 (SAS Institute, Cary, US).

## Results

Until 31 August 2021, more than 101 million vaccine doses had been administered as part of the COVID-19 immunisation programme in Germany, including almost 77 million doses Comirnaty, > 9 million vaccinations Spikevax, > 12 million doses Vaxzevria and > 2.8 million doses COVID-19 Vaccine Janssen. From 1 January 2020 through 31 March 2021, > 15.5 million doses of influenza vaccines had been administered in Germany.

Between 27 December 2020 and 31 August 2021, 214 cases of GBS following COVID-19 vaccination were reported (Figure). Of those, we excluded 46 with TTO < 3 days, > 42 days or unknown [14]. We excluded cases that occurred 1–2 days after vaccination because, for biological reasons (implausible time to onset), it is more likely that these cases can be attributed to other causes and can be considered coincidental. A further 12 cases were excluded because of any unspecific clinical (e.g. upper respiratory, gastrointestinal) or laboratory evidence (e.g. *Campylobacter jejuni*) of antecedent infectious illness occurred within 6 weeks before onset of first neurological symptoms.

**Figure:**
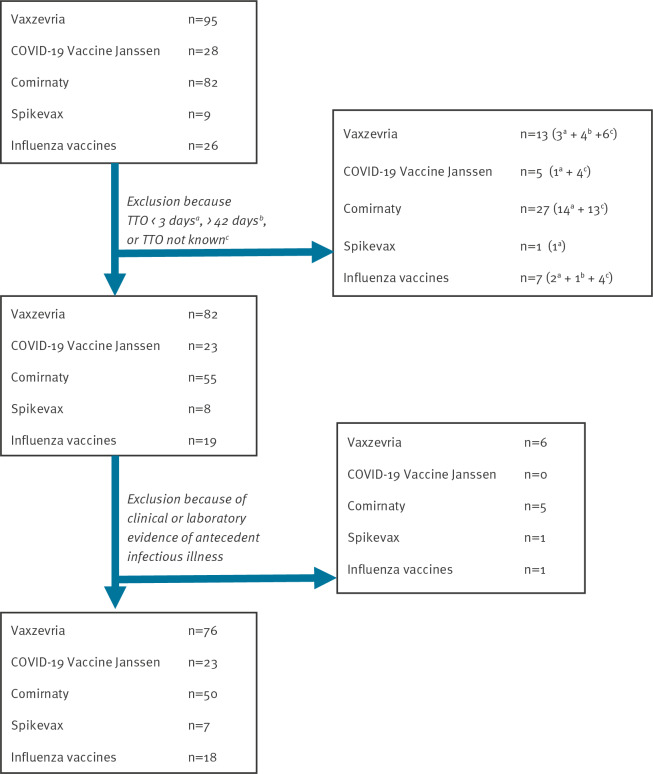
Flow diagram of reported cases with Guillain-Barré syndrome following COVID-19 or influenza vaccination, Germany, 27 December 2020–31 August 2021 (n = 240)

TTO: time to onset.

With the exception of COVID-19 Vaccine Janssen, all vaccines were recommended as a 2-dose vaccination schedule. The second dose should be administered 21 or 28 days after the first dose and no later than 42 days. Cases of GBS related to Vaxzevria were predominantly reported after the administration of the first dose (n = 59 after first dose, n = 4 after second dose; n = 13 dose unknown).

No signal for GBS was detected following vaccination with any influenza vaccine. Seven of 26 reviewed GBS cases following vaccination with any influenza vaccine were excluded because TTO was < 3 days, > 42 days or unknown, and one case was excluded because of clinical or laboratory evidence of antecedent infectious illness.

The clinical characteristics of the analysed 156 GBS cases following COVID-19 vaccination and 18 cases following influenza vaccination are shown in Table 1.

**Table 1:** Demographic characteristics of cases with Guillain-Barré syndrome 3–42 days after vaccination with COVID-19 or influenza vaccines, Germany, 27 December 2020–31 August 2021 (n = 174)

Characteristics	Vaxzevria		COVID-19 Vaccine Janssen			Comirnaty			Spikevax			Influenza vaccines		
	n	%	n		%	n		%	n		%	n		%
Total cases (n)	76		23			50			7			18		
Total GBS cases (n)	72		23			47			6			18		
Total MFS cases (n)	4		0			3			1			0		
Reporting rate (n cases/1,000,000 doses)	6.01		8.06			0.65			0.74			1.16		
Sex														
Female	38	50.0	6	26.1		21	42.0		2	28.6		14	77.8	
Male	38	50.0	16	69.6		29	58.0		5	71.4		4	22.2	
Unknown	0	0.0	1	4.3		0	0.0		0	0.0		0	0.0	
Age group (years)														
≤ 19	0	0.0	0	0.0		1	2.0		0	0.0		0	0.0	
20–29	3	3.9	1	4.3		4	8.0		0	0.0		0	0.0	
30–39	7	9.2	1	4.3		10	20.0		0	0.0		0	0.0	
40–49	13	17.1	4	17.4		4	8.0		1	14.3		1	5.6	
50–59	22	28.9	6	26.1		10	20.0		0	0.0		5	27.8	
60–69	17	22.4	9	39.1		9	18.0		2	28.6		3	16.7	
70–79	12	15.8	0	0.0		7	14.0		2	28.6		2	11.1	
≥ 80	1	1.3	2	8.7		5	10.0		2	28.6		3	16.7	
Unknown	1	1.3	0	0.0		0	0.0		0	0.0		4	22.2	
Mean age (years)	55.5		56.9			54.8			69.1			65.4		
Facial paresis														
Yes	22	28.9	9	39.1		4	8.0		0	0.0		1	5.6	
Bilateral	15	19.7	6	26.1		3	6.0		0	0.0		0	0.0	
Unilateral	7	9.2	3	13.0		1	2.0		0	0.0		1	5.6	
FDP variant														
Yes	1	1.3	2	8.7		0	0.0		0	0.0		0	0.0	
BC level														
1 All GBS	32	42.1	12	52.2		10	20.0		1	14.3		2	11.1	
1 MFS	0	0.0	0	0.0		1	2.0		0	0.0		0	0.0	
2 All GBS	9	11.8	0	0.0		6	12.0		1	14.3		0	0.0	
2 MFS	0	0.0	0	0.0		1	2.0		0	0.0		0	0.0	
3 All GBS	3	3.9	2	8.7		3	6.0		0	0.0		0	0.0	
3 MFS	0	0.0	0	0.0		0	0.0		0	0.0		0	0.0	
4 All GBS	32	42.1	9	39.1		31	62.0		5	71.4		16	88.9	
4 MFS	4	5.3	0	0.0		1	2.0		1	14.3		0	0.0	
Time to onset														
Mean time to onset (days)	14.8		17.6			14.4			14.3			13.9		
Mean time to onset in cases with facial paresis (days)	15.0		16.8			12			NA			26		
Outcome^a^														
Recovered	1	1.3	3	13.0		1	2.0		0	0.0		0	0.0	
Recovering/resolving	16	21.1	5	21.7		9	18.0		1	14.3		0	0.0	
Not recovered/not resolved	57	75.0	12	52.2		39	78.0		6	85.7		16	88.9	
Recovered/resolved + sequelae	0	0.0	1	4.3		0	0.0		0	0.0		0	0.0	
Unknown	0	0.0	2	8.7		0	0.0		0	0.0		2	11.1	
Fatal	2	2.6	0	0.0		1	2.0		0	0.0		0	0.0	

BC: Brighton criteria; FDP: facial diplegia and paraesthesia; MFS: Miller-Fisher syndrome; GBS: Guillain-Barré syndrome; NA: not applicable.

^a^ Reported outcome at the time of reporting or last follow up-information. Fatal outcome was in the course of GBS.

Patients who developed GBS after vaccination with Vaxzevria and COVID-19 Vaccine Janssen frequently had facial paresis (22 of 76 and nine of 23 respectively) compared with Comirnaty (four of 50), Spikevax (0 of seven) and influenza vaccines (one of 18). Concerning patients vaccinated with Vaxzevria and COVID-19 Vaccine Janssen, bilateral facial paresis was reported in 19.7% and 26.1%, whereas the FDP variant (without significant paresis) was only reported in 1.3% and 8.7%.

The clinical course of GBS patients with facial paresis in terms of severity, duration, response to therapy, outcome or the mean time to onset of symptoms was not recognisably different from the other GBS patients without facial paresis after vaccination with Vaxzevria, COVID-19 Vaccine Janssen and influenza vaccines (data not shown for the latter).

### Observed vs expected analysis

The results of the OvE analysis based on GBS cases who fulfilled the criteria of BC levels of diagnostic certainty 1 to 4 are presented in Table 2.

**Table 2:** Observed vs expected analysis for Guillain-Barré syndrome stratified by vaccine and age group (risk window 3–42 days), Germany, 27 December 2020–31 August 2021 (n = 174)

Age group (years)	Background-incidence (cases per 100,000 per year)		Vaxzevria			COVID-19 Vaccine Janssen			Comirnaty			Spikevax			Influenza vaccines		
	Point estimate	95% CI	Cases (n)	SMR	95% CI	Cases (n)	SMR	95% CI	Cases (n)	SMR	95% CI	Cases (n)	SMR	95% CI	Cases (n)	SMR	95% CI
≤ 29^a^	1.10	0.99–1.23	3	2.57	0.53–7.52	1	1.51	0.04–8.43	5	0.40	0.13–0.93	0	NA		0	NA	
30–39	1.52	1.36–1.69	7	3.95	1.59–8.14	1	1.17	0.03–6.53	10	0.67	0.32–1.23	0	NA		0	NA	
40–49	1.30	1.16–1.46	13	7.28	3.88–12.45	4	5.55	1.51–14.21	4	0.29	0.08–0.75	1	0.48	0.01–2.68	1	0.63	0.02–3.52
50–59	2.11	1.91–2.31	22	4.38	2.75–6.64	6	3.77	1.38–8.20	10	0.29	0.14–0.54	0	NA		5	0.93	0.30–2.17
60–69	2.76	2.51–3.02	17	1.35	0.79–2.17	9	6.76	3.09–12.82	9	0.24	0.11–0.46	2	0.53	0.06–1.91	3	0.28	0.06–0.82
70–79	2.80	2.50–3.13	12	1.57	0.81–2.74	0	NA		7	0.21	0.08–0.43	2	0.77	0.09–2.79	2	0.20	0.02–0.71
≥ 80^b^	2.36	1.97–2.81	1	0.72	0.02–4.01	2	16.82	2.04–60.77	5	0.19	0.06–0.44	2	1.56	0.19–5.62	3	0.40	0.08–1.18
Age unknown	NA		1	NA		0	NA		0	NA		0	NA		4	NA	
**Total**	**1.77**	**1.70–1.84**	**76**	**3.10**	**2.44–3.88**	**23**	**4.16**	**2.64–6.24**	**50**	**0.34**	**0.25–0.44**	**7**	**0.38**	**0.15–0.79**	**18**	**0.60**	**0.35–0.94**

CI: confidence interval; GBS: Guillain-Barré syndrome; NA: not applicable; SMR: standardised morbidity ratio.

Background incidence after [18] based on 2,319 adult GBS cases from 1987 to 2016 in Denmark, 1,348 males (58.1%) and 971 females (41.9%).

^a^ Point estimate, 95% CI referring to patients aged 16–29 years.

^b^ Point estimate, 95% CI referring to patients aged 80–89 years.

For all age groups, the SMR for Vaxzevria and COVID-19 Vaccine Janssen were, respectively, 3.10 (95% CI: 2.44–3.88) and 4.16 (95% CI: 2.64–6.24) in the 3–42-day window. After administration of Vaxzevria, significantly elevated SMR were obtained for the age groups 30–39 years, 40–49 years and 50–59 years. With respect to COVID-19 Vaccine Janssen, the following age groups had significantly elevated SMR: 40–49 years, 50–59 years, 60–69 years and 80–89 years. 

No increased SMR were obtained for mRNA COVID-19 vaccines and influenza vaccines.

For a sensitivity analysis, we included only cases who fulfilled the criteria of BC levels 1 to 3 of diagnostic certainty (Table 3). Increased SMR were found particularly in the middle-aged population vaccinated with either Vaxzevria or COVID-19 Vaccine Janssen.

**Table 3:** Observed vs expected analysis for Guillain-Barré syndrome (BC levels 1 to 3) stratified by vaccine and age group (risk window 3 to 42 days), Germany, 27 December 2020–31 August 2021 (n = 81)

Age group (years)	Background-incidence (cases per 100,000 per year)		Vaxzevria			COVID-19 Vaccine Janssen			Comirnaty			Spikevax			Influenza vaccines		
	Point estimate	95% CI	Cases (n)	SMR	95% CI	Cases (n)	SMR	95% CI	Cases (n)	SMR	95% CI	Cases (n)	SMR	95% CI	Cases (n)	SMR	95% CI
≤ 29^a^	1.10	0.99–1.23	2	1.72	0.21–6.20	0	NA		2	0.16	0.02–0.58	0	NA		0	NA	
30–39	1.52	1.36–1.69	3	1.69	0.35–4.95	1	1.17	0.03–6.53	2	0.13	0.02–0.48	0	NA		0	NA	
40–49	1.30	1.16–1.46	7	3.92	1.58–8.08	2	2.77	0.34–10.02	2	0.15	0.02–0.53	0	NA		0	NA	
50–59	2.11	1.91–2.31	15	2.99	1.67–4.93	3	1.88	0.39–5.51	3	0.09	0.02–0.26	0	NA		0	NA	
60–69	2.76	2.51–3.02	12	0.95	0.49–1.67	6	4.50	1.65–9.80	3	0.08	0.02–0.24	1	0.26	0.01–1.47	0	NA	
70–79	2.80	2.50–3.13	5	0.65	0.21–1.53	0	NA		3	0.09	0.02–0.26	0	NA		1	0.10	0.002–0.55
≥ 80^b^	2.36	1.97–2.81	0	NA		2	16.82	2.04–60.77	4	0.15	0.04–0.39	1	0.78	0.02–4.33	1	0.13	0.003–0.75
**Total**	**1.77**	**1.70–1.84**	**44**	**1.79**	**1.30–2.41**	**14**	**2.53**	**1.38–4.25**	**19**	**0.13**	**0.08–0.20**	**2**	**0.11**	**0.01–0.40**	**2**	**0.07**	**0.01–0.24**

CI: confidence interval; GBS: Guillain-Barré syndrome; NA: not applicable; SMR: standardised morbidity ratio.

Background incidence after [18]) based on 2,319 adult GBS cases from 1987 to 2016 in Denmark, 1,348 males (58.1%) and 971 females (41.9%).

^a^ Point estimate, 95% CI referring to patients aged 16–29 years.

^b^ Point estimate, 95% CI referring to patients aged 80–89 years.

The sensitivity analyses using two shorter risk windows (3–14 days and 3–30 days) had results similar to the primary analysis with slightly higher SMR point estimates. We provide the detailed results in the Supplement.

## Discussion

The numbers of observed GBS cases with symptom onset within 3–42 days after receipt of the vector-based COVID-19 vaccines Vaxzevria and COVID-19 Vaccine Janssen exceeded the expected number of cases by a factor of 3.1 and 4.16, respectively. In contrast, neither vaccination with an mRNA COVID-19 vaccine nor vaccination with an influenza vaccine was associated with a higher-than-expected number of reported GBS cases. These data provide supplemental evidence that, although rare, GBS may be an adverse event following vaccination with Vaxzevria or COVID-19 Vaccine Janssen. Our findings are in line with a study from India [3], retrospective analyses of electronic health records and immunisation databases in England [19,20] and a self-controlled case series study from the UK that found a 2.9-fold increased risk of GBS with Vaxzevria, but not with Comirnaty [5]. Similarly, an OvE analysis of GBS cases reported to the US Vaccine Adverse Event Reporting System between February 2021 until July 2021 found a similar increased number of expected GBS cases who received COVID-19 Vaccine Janssen (4.18 for 18–65 years-olds) [21]. Likewise, a cohort study based on data of a Vaccine Safety Datalink in the US found an increased incidence of GBS compared with the background incidence, 21 days after vaccination with COVID-19 Vaccine Janssen but not with Comirnaty or Spikevax [22]. 

In contrast to the reports of Maramattom et al., Walker et al. and Keh et al. [3,19,20], we applied an established signal detection method to assess an uncommon signal for GBS in defined age populations for all four COVID-19 vaccines (including two vector-based vaccines) and influenza vaccines available at the time. We ensured rigorous case ascertainment by analysis of medical records (including laboratory and electrophysiological parameters) and, in case of insufficient data, follow-up information was requested from the reporting physician. Our approach of case ascertainment therefore confirms and adds to studies that identified GBS by use of a hospital statistics database or medical health records [5].

Possible limitations of our study are that a passive surveillance system as it was used here is prone to biases such as underreporting, stimulated reporting, selective reporting and paucity of information [23]. Limitations of the OvE analysis include varying background incidence data in the literature, reporting delays and shorter follow-up intervals for the most recently administered doses as the cut-off date for vaccinations and case reports was 31 August 2021. Further limitations of our study include that we used a GBS background rate from another country (Denmark) and from before the COVID-19 pandemic, and that the data could not be stratified according to potential confounding factors such as sex or preference to certain vaccine products in some age groups. In addition, statistical power was limited for influenza vaccines, as the small overall numbers allowed a signal to be detected for ‘any’ influenza vaccine but precluded this analysis for individual products. The observation period for influenza vaccines was during the COVID-19 pandemic, which may constitute a potential limitation because the data might not be comparable to the time before of the pandemic as COVID-19 itself might cause GBS. We tried, as far as possible, to use the same observation periods for GBS after COVID-19 vaccination and influenza vaccination to avoid comparison with historical data. Until the end of August 2021, the original severe acute respiratory syndrome coronavirus 2 Wuhan virus and the Beta and Delta variants prevailed. The Omicron variant, if any, was not yet common, so most COVID-19 disease was manifest and not latent. In the hospitals, all patients were tested for COVID-19 on admission. When GBS is suspected, the patient is usually tested for a range of potential pathogens and the patient's medical history is searched for potential triggers for GBS. On balance, we do not expect any relevant confounding from COVID-19 vaccination or infection.

The second aim of our study was to assess whether facial paresis, or the FDP variant, as defined by Susuki et al. and Wakerley and Yuki occurred more frequently in GBS cases following COVID-19 vaccination [9,10]. We did not find an increased frequency of the FDP variant after vaccination with vector-based vaccines. We found that bilateral facial paresis was more frequent in GBS cases following vaccination with Vaxzevria and COVID-19 Vaccine Janssen, but not the other vaccines. Bilateral facial paresis occurs usually in less than 5% of all GBS cases [12]. A higher frequency of bilateral facial paresis in GBS following vaccination with vector-based vaccines was also reported by Maramattom et al. and Allen et al., who described this clinical phenotype following vaccination with Vaxzevria [3,4]. The reason for the phenotypic difference is unknown, but clinical variants of GBS can be attributed to differences in the distribution of potential autoantigens, i.e. gangliosides. For example, ganglioside GQ1b is highly expressed in cranial nerves that innervate the extraocular muscles. Patients with MFS express anti-GQ1b antibodies and develop ophthalmoplegia. It is conceivable that yet unknown antibody targets may be present in the facial nerve and that those are relevant for the development of post-infectious and post-vaccination GBS associated with bilateral weakness.

## Conclusion

Our data indicate that although rare, GBS is an adverse event associated with vector-based but not mRNA vaccines. This potential small risk does not outweigh the immense benefits that the vaccination has already demonstrated in the COVID-19 pandemic. Moreover, GBS that occurs after vaccination with Vaxzevria and COVID-19 Vaccine Janssen may show phenotypic differences with an increased frequency of bilateral facial paresis. Potential implications could be that post-vaccine GBS may have a different pathogenesis, and that in case of confounding aetiologies, e.g. co-occurring infections, the absence of bilateral weakness may make a post-vaccination GBS less likely.

## Supplementary Data

164000 Supplement
