# Supplementary material for: Rare cases of Guillain-Barré syndrome after COVID-19 vaccination, Germany, December 2020 to August 2021
Source: Euro Surveill. 2023 Jun 15;28(24):2200744. doi: 10.2807/1560-7917.ES.2023.28.24.2200744 (PMC10318936; doi:10.2807/1560-7917.ES.2023.28.24.2200744)
Supplement: Supplement [file 22-00744_STREIT_Supplement.pdf]

This supplementary material is hosted by *Eurosurveillance* as supporting information alongside the article „Rare cases of Guillain-Barré syndrome after COVID-19 vaccination, Germany, December 2020 to August 2021“, on behalf of the authors, who remain responsible for the accuracy and appropriateness of the content. The same standards for ethics, copyright, attributions and permissions as for the article apply. Supplements are not edited by *Eurosurveillance* and the journal is not responsible for the maintenance of any links or email addresses provided therein.

Observed vs expected analysis for Guillain-Barré syndrome stratified by vaccine and age group (risk window 3–14 and 3-30 days), Germany, 27 December 2020–31 August 2021 (n = 105 for 3-14days; n = 163 for 3-30days)

Age group (years)	Background-incidence (cases per 100,000 per year)		Time to onset	Vaxzevria			Covid-19 Vaccine Janssen			Comirnaty			Spikevax			Influenza vaccines		
	Point estimate	95% CI		Cases (n)	SMR	95% CI	Cases (n)	SMR	95% CI	Cases (n)	SMR	95% CI	Cases (n)	SMR	95% CI	Cases (n)	SMR	95% CI
≤29[a]	1.10	(0.99–1.23)	14	1	2.86	(0.07- 15.93)	1	5.04	(0.13- 28.11)	3	0.80	(0.16- 2.33)	0	NA		0	NA	
			30	3	3.68	(0.76- 10.74)	1	2.16	(0.05- 12.05)	4	0.46	(0.12- 1.17)	0			0		
30-39	1.52	(1.36–1.69)	14	4	7.52	(2.05- 19.26)	0	NA		9	2.01	(0.92- 3.81)	0			0		
			30	7	5.64	(2.27- 11.62)	1	1.67	(0.04- 9.33)	10	0.96	(0.46- 1.76)	0			0		
40-49	1.30	(1.16–1.46)	14	5	9.33	(3.03- 21.78)	2	9.25	(1.12- 33.41)	1	0.24	(0.01- 1.36)	1	1.60	(0.04- 8.93)	0		
			30	13	10.40	(5.54- 17.78)	4	7.93	(2.16- 20.30)	4	0.42	(0.11- 1.07)	1	0.69	(0.02- 3.83)	1	0.90	(0.023-5.02)
50-59	2.11	(1.91–2.31)	14	14	9.30	(5.08- 15.60)	4	8.37	(2.28- 21.44)	4	0.39	(0.11- 1.01)	0	NA		4	2.48	(0.67-6.34)
			30	20	5.69	(3.48- 8.79)	5	4.49	(1.46- 10.47)	6	0.25	(0.09- 0.55)	0			5	1.33	(0.43-3.10)
60-69	2.76	(2.51–3.02)	14	12	3.18	(1.64- 5.56)	2	5.00	(0.61- 18.08)	8	0.72	(0.31- 1.42)	1	0.88	(0.02- 4.90)	3	0.94	(0.19-2.74)
			30	17	1.93	(1.13- 3.09)	9	9.65	(4.41- 18.32)	9	0.35	(0.16- 0.66)	2	0.75	(0.09- 2.73)	3	0.40	(0.08-1.18)
70-79	2.80	(2.50–3.13)	14	6	2.62	(0.96- 5.70)	0	NA		3	0.30	(0.06- 0.87)	1	1.29	(0.03- 7.18)	2	0.66	(0.08-2.37)
			30	11	2.06	(1.03- 3.68)	0			6	0.26	(0.09- 0.56)	1	0.55	(0.01- 3.08)	2	0.28	(0.03-1.01)
80+[b]	2.36	(1.97–2.81)	14	1	2.40	(0.06- 13.35)	1	28.04	(0.71- 156.22)	5	0.63	(0.20- 1.47)	1	2.59	(0.07- 14.45)	2	0.89	(0.11-3.23)
			30	1	1.03	(0.03- 5.72)	2	24.03	(2.91- 86.82)	5	0.27	(0.09- 0.63)	2	2.22	(0.27- 8.03)	3	0.58	(0.12-1.68)
Age unknown	NA		14	1	NA		0	NA		0	NA		0	NA		3	NA	
			30	1			0			0			0			4		
Total	1.77	(1.70–1.84)	14	44	5.98	(4.35- 8.03)	10	6.03	(2.89- 11.09)	33	0.74	(0.51- 1.04)	4	0.73	(0.20- 1.87)	14	1.55	(0.85-2.60)
			30	73	4.25	(3.33- 5.35)	22	5.68	(3.56- 8.61)	44	0.42	(0.31- 0.57)	6	0.47	(0.17- 1.02)	18	0.85	(0.51-1.35)

CI: confidence interval; GBS: Guillain-Barré syndrome; NA: not applicable; SMR: standardised morbidity ratio.

Background incidence after [17] based on 2,319 adult GBS cases from 1987 to 2016 in Denmark, 1,348 males (58.1%) and 971 females (41.9%).

[a] Point estimate, 95% CI referring to patients aged 16–29 years.

[b] Point estimate, 95% CI referring to patients aged 80–89 years.

Observed vs expected analysis for Guillain-Barré syndrome (BC levels 1 to 3) stratified by vaccine and age group (risk window 3-14 and 3-30 days), Germany, 27 December 2020–31 August 2021 (n = 49 for 3-14days; n = 78 for 3-30days)

Age group (years)	Background-incidence (cases per 100,000 per year)		Time to onset	Vaxzevria			Covid-19 Vaccine Janssen			Comirnaty			Spikevax			Influenza vaccines		
	Point estimate	95% CI		Cases (n)	SMR	95% CI	Cases (n)	SMR	95% CI	Cases (n)	SMR	95% CI	Cases (n)	SMR	95% CI	Cases (n)	SMR	95% CI
≤29[a]	1.10	(0.99–1.23)	14	1	2.86	(0.07- 15.93)	0	NA		1	0.27	(0.01-1.48)	0	NA		0	NA	
			30	2	2.45	(0.30- 8.85)	0			1	0.11	(0.003-0.64)	0			0		
30-39	1.52	(1.36–1.69)	14	2	3.76	(0.46-13.59)	0			2	0.45	(0.05-1.61)	0			0		
			30	3	2.42	(0.50-7.07)	1	1.67	(0.04-9.33)	2	0.19	(0.02-0.69)	0			0		
40-49	1.30	(1.16–1.46)	14	3	5.60	(1.15-16.37)	2	9.25	(1.12-33.41)	1	0.24	(0.01-1.36)	0			0		
			30	7	5.60	(2.25-11.54)	2	3.96	(0.48-14.32)	2	0.21	(0.03-0.76)	0			0		
50-59	2.11	(1.91–2.31)	14	9	5.98	(2.73-11.35)	2	4.19	(0.51-15.12)	2	0.20	(0.02-0.71)	0			0		
			30	14	3.99	(2.18-6.69)	3	2.69	(0.56-7.87)	2	0.08	(0.01-0.30)	0			0		
60-69	2.76	(2.51–3.02)	14	8	2.12	(0.92-4.18)	1	2.50	(0.06-13.94)	2	0.18	(0.02-0.65)	1	0.88	(0.02-4.90)	0		
			30	12	1.36	(0.70-2.38)	6	6.43	(2.36-14.00)	3	0.12	(0.02-0.34)	1	0.38	(0.01-2.10)	0		
70-79	2.80	(2.50–3.13)	14	2	0.87	(0.11-3.15)	0	NA		3	0.30	(0.06-0.87)	0	NA		1	0.33	0.008-1.83
			30	5	0.94	(0.30-2.18)	0			3	0.13	(0.03-0.37)	0			1	0.14	0.004-0.78
80+[b]	2.36	(1.97–2.81)	14	0	NA		1	28.04	(0.71-156.22)	4	0.50	(0.14-1.29)	0			1	0.45	0.011-2.49
			30	0			2	24.03	(2.91-86.82)	4	0.22	(0.06-0.55)	1	1.11	(0.03-6.19)	1	0.19	0.005-1.07
Age unknown	NA		14	0			0	NA		0	NA		0	NA		0	NA	
			30	0			0			0			0			0		
Total	1.77	(1.70–1.84)	14	25	3.40	(2.20-5.02)	6	3.62	(1.33-7.87)	15	0.34	(0.19-0.55)	1	0.18	(0.01-1.02)	2	0.22	0.027-0.80
			30	43	2.51	(1.81-3.38)	14	3.62	(1.98-6.07)	17	0.16	(0.09-0.26)	2	0.16	(0.02-0.57)	2	0.09	0.011-0.34

CI: confidence interval; GBS: Guillain-Barré syndrome; NA: not applicable; SMR: standardised morbidity ratio.

Background incidence after [17] based on 2,319 adult GBS cases from 1987 to 2016 in Denmark, 1,348 males (58.1%) and 971 females (41.9%).

[a] Point estimate, 95% CI referring to patients aged 16–29 years.

[b] Point estimate, 95% CI referring to patients aged 80–89 years.
